# Larvae and Nests of Six Aculeate Hymenoptera (Hymenoptera: Aculeata) Nesting in Reed Galls Induced by *Lipara* spp. (Diptera: Chloropidae) with a Review of Species Recorded

**DOI:** 10.1371/journal.pone.0130802

**Published:** 2015-06-26

**Authors:** Petr Bogusch, Alena Astapenková, Petr Heneberg

**Affiliations:** 1 Department of Biology, Faculty of Science, University of Hradec Králové, Hradec Králové, Czech Republic; 2 Third Faculty of Medicine, Charles University in Prague, Praha, Czech Republic; Institute of Zoology, CHINA

## Abstract

Wetland species of aculeate Hymenoptera are poorly known, even though many of them may serve as diagnostic or flagship species in nature conservation. Here we examined 6,018 galls induced ≥1 year prior their collection by the chloropid flies *Lipara* spp. The galls were collected at 34 sites in Central Europe. We examined 1,389 nests (4,513 individuals) of nine species, part of which were parasitized by one dipteran and two chrysidid parasitoid species. We describe the nests of seven dominant species and larvae of four species (*Pemphredon fabricii*, *Trypoxylon deceptorium*, *Hoplitis leucomelana *and *Hylaeus pectoralis*) and two parasitoids (*Trichrysis cyanea* and *Thyridanthrax fenestratus*, both in nests of *Pemphredon fabricii *and *Trypoxylon deceptorium*). All the species, but *H*. *pectoralis*, preferred robust galls at very thin stalks (induced typically by *Lipara lucens*) over the narrow galls on thick stalks. The larvae of *P*. *fabricii* and *T*. *deceptorium *resembled strongly their sibling species (*Pemphredon lethifer *and *Trypoxylon attenuatum* sensu lato, respectively). The larvae of *T*. *fenestratus* showed features different from those previously described. By hatching set of another 10,583 galls induced by *Lipara* spp. ≥1 year prior their collection, we obtained 4,469 individuals of 14 nesting hymenopteran species, two cleptoparasites, three chrysidid and one dipteran parasitoid. Of these species, four new nesting species have been recorded for the first time in galls induced by *Lipara* spp.: *Chelostoma campanularum*, *Heriades rubicola*, *Pseudoanthidium lituratum* and *Hylaeus incongruus*. We also provide first records of their nest cleptoparasites *Stelis breviuscula* and *Stelis ornatula*, and the parasitoid *Holopyga fastuosa generosa*. *Thyridanthrax fenestratus* formed strong populations in nests of *Pemphredon fabricii* and *Trypoxylon deceptorium*, which are both newly recorded hosts for *T*. *fenestratus*. The descriptions provided here allow for the first time to identify the larvae of the most widespread central European aculeate hymenopteran reed gall specialists.

## Introduction

Cavity-nesting Hymenoptera developed a wide range of strategies allowing them to use a broad spectrum of cavities for nesting. Among the Palearctic species, a specific community of bees and wasps make their nests in the galls of chloropid flies. Most frequently, they use the galls induced by *Lipara lucens* (Chloropidae) on common reed *Phragmites australis* (Poaceae) stems [1–3]. Some of these aculeate hymenopteran species, such as the digger wasp *Pemphredon fabricii* (Crabronidae) or the solitary bee *Hylaeus pectoralis* (Colletidae) are specialized for nesting in galls induced by *Lipara* spp. more than a year ago (old galls) [2–3]. However, most of the other aculeate hymenopteran species found in the reed galls are capable to use many different kinds of cavities for their nests such as cut reed, old larval galleries in wood and cavities in old walls. Combined, the reed galls in central and north Europe are confirmed to host altogether 25 species of aculeate Hymenoptera (superfamilies Chrysidoidea, Vespoidea and Apoidea) [2–4] and 3 species of their parasitoids of the family Chrysididae. Some of these species (e.g., *Pemphredon fabricii*) are locally very abundant, whereas many others (such as *Rhopalum gracile* (Crabronidae)) are extremely rare and considered critically endangered or endangered in the regional red-lists [5–9].

The biology, nest structure and larval morphology are known for only few species of the aculeate hymenopteran reed gall nesters. *Pemphredon fabricii* is the most numerous aculeate hymenopteran species in reed galls [2–3]. This species was for a long time considered to be a form or a subspecies of closely related *Pemphredon lethifer*, which is more widespread and ecologically tolerant. Special blunt claws on tarsi of this species work as a very good adaptation for moving in reed, and this species does not occur in any other habitats than reed beds [10–11]. This species was resurrected from the synonymy [12] and its nesting habits were described [13] together with its specificity to reeds, which was later confirmed by [2–3]. Mature larvae of the related species *P*. *lethifer* were described previously [14–15]. In both cases, the descriptions of the nest and mature larvae were based on the specimens obtained from the hollow stems of *Rubus* spp. (Rosaceae), and thus do not represent the nests and larvae of *P*. *fabricii*.

*Hylaeus pectoralis* is a rare bee, occurring only in wetlands. It is specialized for nesting in old *Lipara* galls, reaching only low population densities at any sites of its presence [2–3]. The species is classified as critically endangered (CR) in the Red List of Czech Invertebrates [9]. However, [3] found that this species only escaped the traditional sampling techniques and was present at multiple sites at least throughout the Czech Republic, thus not being as rare as was previously supposed (cf. [16–17]). Its larvae and nests were described [18], and its biology was briefly described from England [19]. Reed galls host also a smaller species of the same genus, *Hylaeus moricei*, which is less specialized and may utilize also other types of cavities. Larva and nest of this species are so far unknown. *Hoplitis leucomelana* (Megachilidae) is another bee nesting frequently in reed galls. It is a generalist and uses many different cavity types for nesting [17, 20]. The larva of this species was also described [21] as well as its nest in the cavity of *Rubus* spp. [20].

Asís et al. described the nesting biology, nest structure and morphology of mature larva of *Trypoxylon attenuatum* sensu lato (Crabronidae) [22]. This digger wasp was previously thought to be a single species, but later it was divided it to six species [23]. Of them, *T*. *deceptorium* is bound to wetlands and nests in reed galls and cavities. Although [3] found this species as the second most abundant aculeate hymenopteran in reed galls (after *P*. *fabricii*), they found that *T*. *attenuatum* is the only *Trypoxylon* spp. occurring in reed galls. So far, there are no data allowing differential morphological diagnosis between the larvae of *T*. *attenuatum* sensu stricto and *T*. *deceptorium*. *Trypoxylon attenuatum* sensu lato nests may be parasitized by *Trichrysis cyanea* (Chrysididae), and mature larva of *T*. *cyanea* and *T*. *attenuatum* were described by [22]. Among all the parasitoids and nest cleptoparasites, *T*. *cyanea* is most abundant in nests of digger wasps in reed galls. The main reason should be that *T*. *cyanea* displays the least host specificity among European golden wasps, invading nests of plenty bee and wasp species [17, 24–26].

In this study, we examined an extensive set of reed galls and analyzed and broadened the known spectrum of aculeate hymenopteran species associated with the reed galls induced by *Lipara* spp. in the Czech Republic and Slovakia, Central Europe. Using the dataset of individually examined reed galls, we analyzed and described the nesting biology and mature larvae of six species of aculeate Hymenoptera. Particularly important are the first available data on the differences in larval morphology of sibling species *P*. *fabricii* and *P*. *lethifer*, as well as *T*. *attenuatum* and *T*. *deceptorium*.

## Materials and Methods

### Study sites and sampling

**Fig 1:**
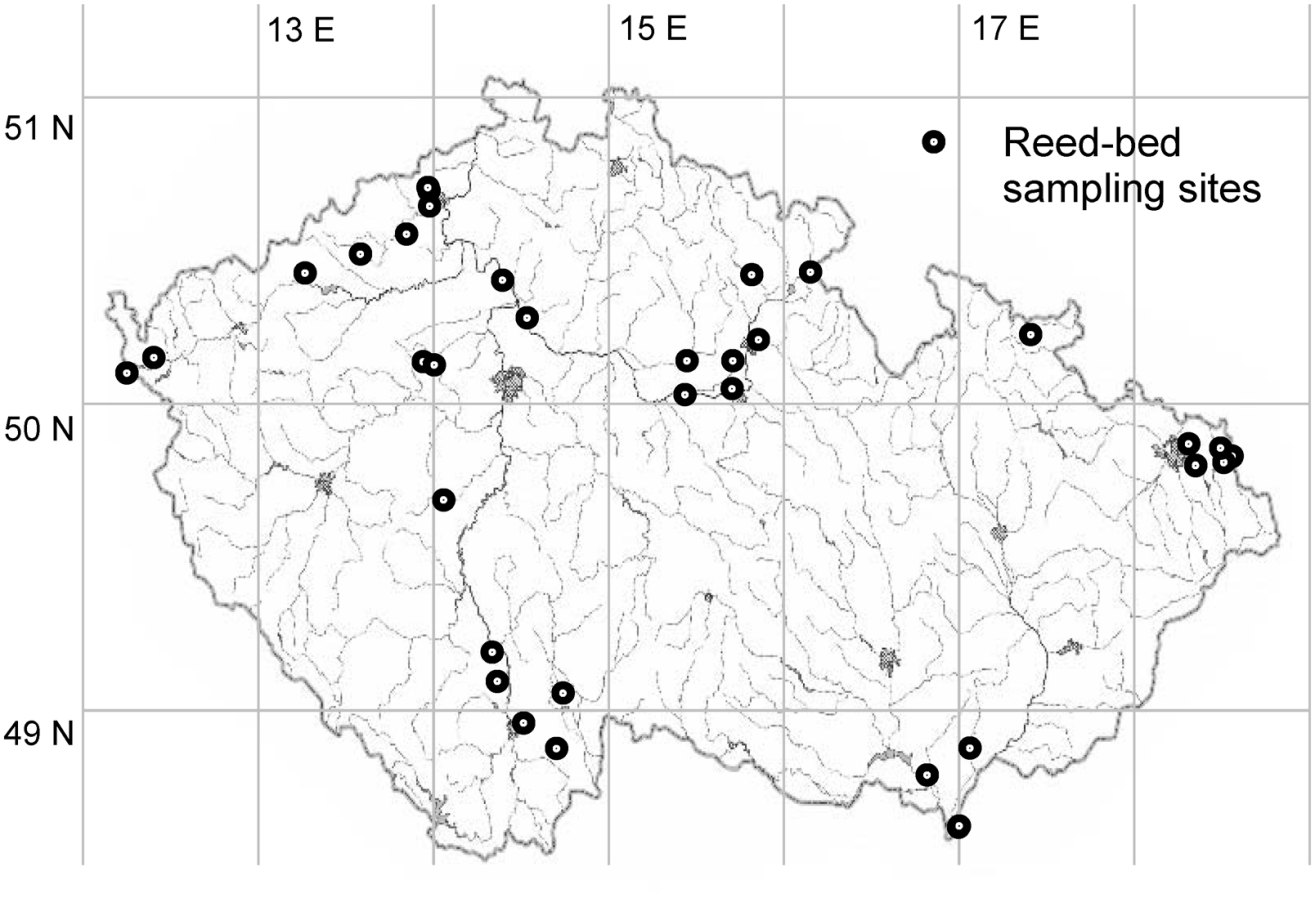
Location of study sites in the Czech Republic and Slovakia.

We have collected galls induced by *Lipara* spp. on common reed at 34 sampling sites located across the Czech Republic (33 sites) and in Slovakia (1 site) (Fig 1), for detailed information see S1 Table. All localities with coordinates are listed in Supporting Information (S1 Table). Study of plants and animals was possible at all localities without any restriction, except the following: Třebeč (Brouskův Mlýn National Nature Reserve (NNR)), permission issued from Blanský les Protected Landscape Area (PLA), personally Jana Janáková, MSc.; Lomnice nad Lužnicí (Velký a Malý Tisý NNR), permission issued from Třeboňsko PLA, personally Dr. Miroslav Hátle; Lednice (Lednické rybníky NNR), permission issued from Pálava PLA, personally Dr. Pavel Dedek; Vonšov (SOOS NNR), permission issued from Slavkovský les PLA, personally Přemysl Tájek; Chvaletice and Trnávka (Power Station Chvaletice fly ash deposits), permission issued from Severní energetická a.s., personally Ing. Karel Polc. The field studies did not involve any animals protected in the Czech Republic or Slovakia and no CITES species.

Only galls older than 1 year (greyish or darker in appearance, usually without leaves and with the apex broken) were collected because of our focus on cavity nesting Hymenoptera (bees and wasps), not on the *Lipara* spp. (inducing the galls) or their parasitoids. We have collected the reed galls in a period from 7 Feb to 23 Mar 2014 and additional material on 4 Sep 2014. In late winter and early spring, the mature larvae are present in their nests, and their rearing is easier than if they would be collected before the hibernation in the autumn months. We collected at least 500 reed galls at each sampling site, of which 200 were longitudinally cut and their contents were analyzed and the rest were allowed to rear. Incidentally collected galls of age <1 year (with *Lipara* spp. or their parasitoids present) were removed from the analyses, thus the total counts of galls analyzed from each site were slightly lower.

### Data acquisition

In the longitudinally cut reed galls, we have studied the material of the walls separating the brood cells (further termed septa) and closing plug at the top of each nest (further termed closing plug), the structure and number of brood cells, and also the morphology and coloration of larvae and pupae. In the description, first cell means the hind, first-built cell of the nest, and last cell means the nearest cell to the nest entrance. When the larvae were in cocoons, we removed part of the larvae out of the cocoons but left the others inside of them. Of each species, we first tried to rear the adults; only for nests with more than three larvae we conserved some brood for morphological studies. From each hymenopteran nest, all living larvae were taken, placed in Eppendorf 1.5 ml micro-tubes, which were closed with cotton wool, left in the laboratory conditions and reared similarly as was described by [27]. The adults usually hatched within three to four weeks after the pupation and then we fixed them (as well as unreared larvae) in 96% ethanol. We measured the maximum diameter of the reed gall and the diameter of the reed stem just below the reed gall.

The reed galls allowed to rear were placed into rearing sacs as described [3], and allowed to hatch for ten weeks. The reared individuals were fixed in an ethylene glycol solution supplemented with a mixture of ionic and anionic detergents and transferred later to the 96% ethanol.

The obtained material was determined by the first author, and representative specimens (including the nests of each species) are available in the collections of University of Hradec Králové (Hradec Králové, Czech Republic). We used the nomenclature according to [16] (aculeate Hymenoptera), [4] (Chloropidae) and [28] (Bombyliidae).

We documented representative part of the nests using a digital camera (photographs of whole nests) and a special macro-photographing apparatus consisting of a macro-camera attached to a stereo microscope (brood cells, whole larvae and pupae). The documentation included the photographs of nests shared by multiple species of aculeate Hymenoptera and of the parasitized nests. We took photos of both, living larvae and the larvae fixed in Pampel solution (30 parts of distilled water, 15 parts of 96% ethanol, 6 of formaldehyde and 4 parts of glacial acetic acid) as described [29]. The photos of living specimens turned to be more suitable for field identification of the here described species. To describe morphology of the larval specimens, we transferred some larvae (but always only a portion of larvae present in a single nest) into the Pampel solution. As soon as we took the photographs of the whole larvae, we focused on their sclerotized parts. For this purpose, we placed the larvae for 12 hours into 10% solution of hot (60°C) potassium hydroxide to dilute all parts of the body except the integument. Then we colored the integument in 5% Chlorazol Black E for 2 seconds and moved the specimens into 96% ethanol for conservation. To observe the identification marks, we placed the integument into glycerol and observed separately the head, mouthparts, spiracles and other important parts of the integument under light microscope. We used the same specimens for the study of very small structures such as setae, sensillae or mouthparts. We drew figures of (1) the head with a focus on the clypeus, labrum, maxillae and labium, (2) the mandibles from anterior view, and (3) the spiracles of each larva.

### Data analysis

The data are shown as means ± SD unless stated otherwise. We analyzed the occupancy rate of three size categories of reed stems and four size categories of reed galls. To analyze the differences between the observed and expected occupancy rates in the particular size categories of reed galls and reed stems, we used χ^2^ tests. The expected frequencies were derived from the frequency of reed galls or stems of the particular diameter within the whole sampled cohort using the following equation:

$$n_{i\;\left(expected\right)}=\frac{n_{i\;\left(observed\right)}}{\sum_{i}n_{\left(observed\right)}}$$

Where *n*_i (expected)_ represents the expected frequency, *n*_i (observed)_ represents the observed frequency of reed galls or stems of the particular diameter within the whole dataset, and Σ_*i*_ *n*_(observed)_ stands for the total number of reed galls or stems collected and measured. To estimate the completeness of the sampled dataset, we computed the rarefaction curve based on the log Gamma function for computing combinatorial terms in PAST v 2.14. To estimate the species richness in the examined dataset, we calculated the Chao-1 estimator, corrected for unseen species in the dataset in EstimateS 9.1.0. Both calculations included all the adult specimens of aculeate Hymenoptera and their parasitoids obtained in course of this study by hatching the imagines from longitudinally cut galls and those reared directly from the galls.

## Results

### Aculeate Hymenoptera in longitudinally cut reed galls of *Lipara* spp.

We found 1389 nests of aculeate Hymenoptera in the total 6,018 longitudinally cut reed galls induced by *Lipara* spp. Of them, we identified 1159 nests of nine species of aculeate Hymenoptera, two species of cuckoo wasps and a single parasitic dipteran species (Table 1). The occupancy rate was highly site-specific, ranging from 66.5% (Chvaletice, PU, Czech Rep.) to zero (two sampling sites Cheb and Vonšov, CH, Czech Rep.) despite we used the identical sampling methodology at all the examined sites. The variability found can be explained by the reed gall parameters as described below.

**Table 1:** Number of galls examined and of those containing the brood of species of aculeate Hymenoptera (Vespoidea, Apoidea) and their parasitoids (Hymenoptera: Chrysidoidea; Diptera: Asiloidea).

Species	Number of individuals reared from intact galls	Relative proportion of individuals reared [%]	Number of nests found in longitudinally cut reed galls	Relative proportion of nests found in reed galls [%]
**Diptera / Bombyliidae**				
*Thyridanthrax fenestratus* (Fallén, 1814)*	44	0.97	18	1.30
**Hymenoptera / Chrysididae**				
*Chrysis angustula* Schenck, 1856*	10	0.22	1	0.07
*Holopyga generosa* Förster, 1853*	1	0.02	0	0.00
*Trichrysis cyanea* (Linnaeus, 1761)*	19	0.42	16	1.15
**Hymenoptera / Vespidae**				
*Symmorphus bifasciatus* (Linnaeus, 1761)	21	0.47	6	0.43
**Hymenoptera / Crabronidae**				
*Ectemnius confinis* (Walker, 1871)	2	0.04	0	0.00
*Nitela spinolai* Latreille, 1809	3	0.07	0	0.00
*Passaloecus clypealis* Faester, 1947	11	0.24	3	0.22
*Pemphredon fabricii* (Müller, 1911)	4205	93.18	1029	74.08
*Rhopalum gracile* Wesmael, 1852	3	0.07	0	0.00
*Trypoxylon deceptorium* Antropov, 1991	38	0.84	39	2.81
*Trypoxylon minus* Beaumont, 1945	6	0.13	6	0.43
**Hymenoptera / Megachilidae**				
*Chelostoma campanularum* (Kirby, 1802)	1	0.02	0	0.00
*Heriades rubicola* Pérez, 1890	3	0.07	0	0.00
*Hoplitis leucomelana* (Kirby, 1802)	16	0.35	10	0.72
*Pseudoanthidium lituratum* (Panzer, 1801)	1	0.02	0	0.00
*Stelis breviuscula* (Nylander, 1848)*	3	0.07	0	0.00
*Stelis ornatula* (Klug, 1807)*	4	0.09	0	0.00
**Hymenoptera / Colletidae**				
*Hylaeus incongruus* Förster, 1871	0	0.00	1	0.07
*Hylaeus moricei* (Friese, 1898)	17	0.38	3	0.22
*Hylaeus pectoralis* Förster, 1871	105	2.33	27	1.94
**Not identified**	0	0.00	230	16.56
**TOTAL (individuals reared)**	**4513**			
**TOTAL (nests found)**			**1389**	
**TOTAL (reed galls examined)**	**10583**		**6449**	

We found 20.6% of one year old reed galls positive for the nests of aculeate Hymenoptera. Indicated are the number of individuals hatched in the rearing bags, number of nests positive for each species found as revealed by longitudinal cutting of the reed galls, and their relative proportions within the total datasets. Species marked by asterisks are parasitic.

*Pemphredon fabricii* was the most abundant species of aculeate Hymenoptera, confirmed in 1012 nests (90% of the non-parasitized nests of aculeate Hymenoptera), and identified at all 34 sampling sites where the hymenopteran nests were present in the collected reed galls. Other aculeate hymenopteran species nested in the galls in smaller numbers. The examined dataset included *Trypoxylon deceptorium* (39 nests found at 10 sampling sites), *Hylaeus pectoralis* (27/9), *Hoplitis leucomelana* (10/6), *Symmorphus bifasciatus* (6/5), *Trypoxylon minus* (6/4), *Hylaeus moricei* (3/3), *Passaloecus clypealis* (3/2) and *Hylaeus incongruus* (1/1). The latter species was recorded for the first time nesting in the reed galls induced by *Lipara* spp.

Among the three species of parasitoids of aculeate Hymenoptera found, the most abundant was the chrysidid wasp *Trichrysis cyanea* identified in 16 nests at 7 sampling sites. As its host species, we confirmed *Pemphredon fabricii* (4 nests containing immature individuals of both species) and *Trypoxylon deceptorium* (1 nest with both species). Additionally, we identified a single nest occupied by *Chrysis angustula* [in a nest of *Pemphredon fabricii* at the sampling site Kamenné Žehrovice (KL, Czech Rep.)]. All the above host-parasitoid associations are new host records (globally). In the nests of *Pemphredon fabricii* at the sampling sites Sekule (SE, Slovakia) and Hodonín (HO, Czech Rep.), we noticed a high parasitation rate by the larvae of bombyliid fly *Thyridanthrax fenestratus*. We identified larvae of this parasitoid in 21 of 89 *P*. *fabricii* nests (24%) at the sampling site Sekule, and reared the *T*. *fenestratus* adults from 18 of them (and also reared the adults of the host species from three of these nests). At the sampling site Hodonín, we identified larvae of *T*. *fenestratus* in 6 of 29 *P*. *fabricii* nests (21%), but the adults did not hatch from any of them. In September 2014, we recorded another *Thyridanthrax fenestratus* individuals in a set of 200 reed galls collected at the sampling site Sekule, where we also found it in cocoons inside one nest of *Trypoxylon deceptorium* (besides *P*. *fabricii*).

**Fig 2:**
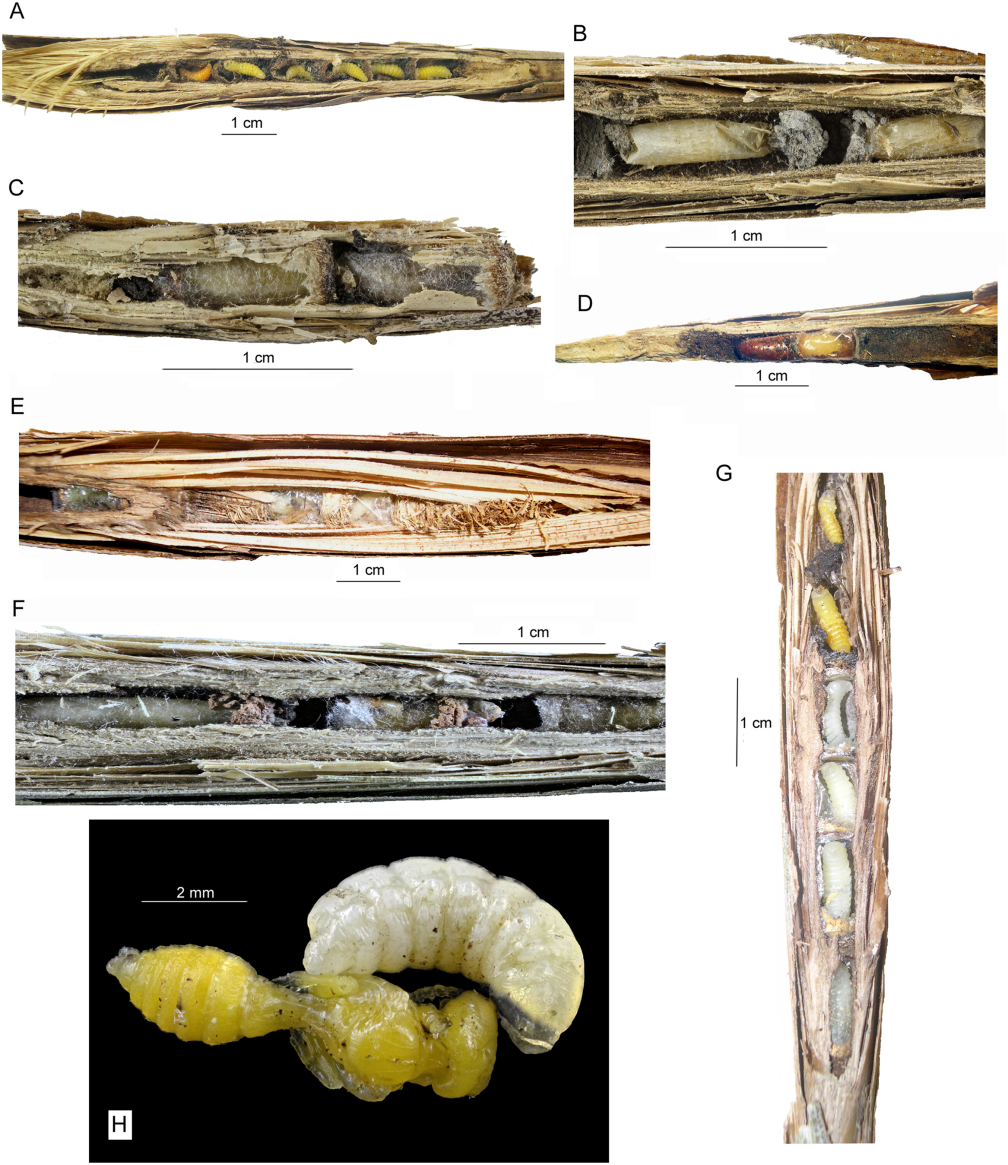
Nests of aculeate Hymenoptera in reed galls of *Lipara* spp. A–*Pemphredon fabricii*, whole nest, B–*Trypoxylon deceptorium*, detail, C–*Hoplitis leucomelana*, detail, D—nest with larva and pupa of *Trichrysis cyanea*, E—*Hylaeus pectoralis*, whole nest, F—parasitized nest of *Trypoxylon deceptorium* with one brood cell of *T*. *deceptorium* and two brood cells of *Trichrysis cyanea*, G—mixed nest of two species containing four white larvae of *Hylaeus pectoralis* (bottom) and two yellow larvae of *Pemphredon fabricii* (top), H—larva of the bombyliid fly *Thyridanthrax fenestratus* on pupa of *Pemphredon fabricii*. All photos by P. Bogusch.

Besides the nests occupied by a single species and those occupied by the parasitoids (see Fig 2A–2F), we found 12 galls containing immature individuals of two different aculeate hymenopteran species. In all cases, the nests were used sequentially (not at the same time): when one species finished its nest, then the second species started to work on its own nest occupying the rest of the space available in the gall. We did not observe any signs of killed brood. The mixed nests included the following combinations: *Pemphredon fabricii* and *Trypoxylon deceptorium* (9×), *Pemphredon fabricii* and *Hylaeus pectoralis* (2×; Fig 2G), and *Pemphredon fabricii* and *Trypoxylon minus* (1×).

### Aculeate Hymenoptera in reared reed galls of *Lipara* spp.

In parallel to the longitudinally cut reed galls, we collected another set of 10,583 reed galls, which were allowed to rear. Their rearing yielded in total 4,469 individuals of 19 species of aculeate Hymenoptera (including 30 individuals of three hymenopteran parasitoid species) and 44 individuals of a single dipteran parasitoid species (Table 1). The species spectrum was similar to that obtained by longitudinal cutting of the collected galls, with some exceptions as specified below. *Pemphredon fabricii* was eudominant among the aculeate hymenopterans with 4,205 adults hatched (94% of the total). The other abundant species included *Hylaeus pectoralis* (91 individuals / 10 sampling sites) and *Trypoxylon deceptorium* (38 individuals / 9 sites). Less than 20 imagines were obtained from the rest of the species found. We reared several species, which were not known as reed gall inquilines so far. Among them were the golden wasp *Holopyga fastuosa generosa* [1 female from Tuchomyšl (UL, Czech Rep.)], the bees *Chelostoma campanularum* [1 female from Dubno (PB, Czech Rep.)], *Heriades rubicola* [3 individuals from Hodonín (HO, Czech Rep.)], *Pseudoanthidium lituratum* [1 male from Hodonín (HO, Czech Rep.)], *Stelis breviuscula* [3 individuals from spoil heap near Srnojedy (PU, Czech Rep.)] and *S*. *ornatula* [4 specimens: 1 from Stonava (KI), 2 from Stará Pohůrka (CB) and 1 from Tuchomyšl (UL, all Czech Rep.)]. The latter two bee species are cleptoparasitic; *S*. *breviuscula* parasitizes *Heriades* spp., and *S*. *ornatula* utilizes *Hoplitis* spp., including *H*. *leucomelana*, as its host species [17]. We also confirmed the presence of two digger wasp species reported earlier by [3]: *Nitela spinolae* at two ash deposits near Pardubice (PU, Czech Rep.) and *Rhopalum gracile* (3 specimens found in Náchod, NA, Czech Rep.), and another digger wasp species reported earlier by [4]: *Ectemnius confinis* [2 males from Doubrava u Orlové (KI, Czech Rep.)]. Regarding the single dipteran parasitoid of aculeate hymenopterans found, *Thyridanthrax fenestratus*, in total 44 imagines hatched from galls collected at the same sampling sites at which we found it in the longitudinally cut galls (21 and 23 individuals, respectively).

**Fig 3:**
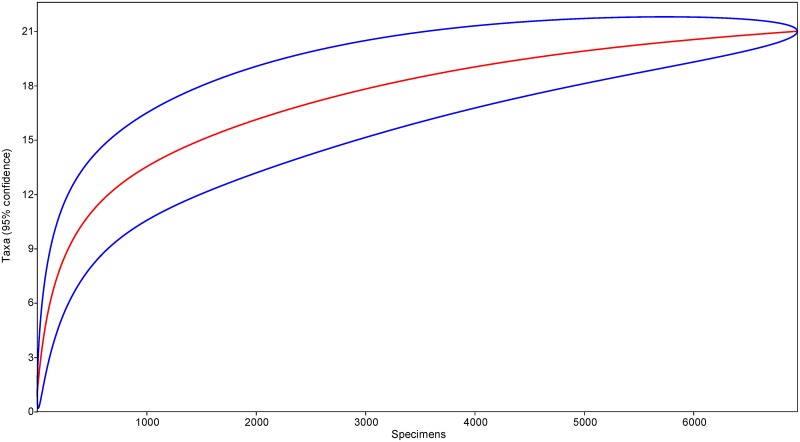
Rarefaction curve of the aculeate Hymenoptera nesting in reed galls and of their parasitoids based on all the hatched adult imagines obtained in course of this study (n = 6,951). Numbers on the X axis indicate the number of individuals hatched (specimens).

In course of this study, we hatched 6,951 adult imagines of aculeate Hymenoptera and their parasitoids, representing 21 species (Table 1). Rarefaction of the obtained dataset (Fig 3) suggested that we reached high level of completeness. The Chao-1 estimator reached 22.0 ± 1.8 (95% CI 21.1–31.7) species. Combined, the total number of species of aculeate Hymenoptera known from galls induced by *Lipara* spp. in Europe now reached 29, including six hymenopteran parasitoids. In addition, we provided the evidence on the presence of a single dipteran parasitoid species of aculeate Hymenoptera in the examined reed galls (Table 1).

### Structure of nests

We analyzed the nests of six species nesting in reed galls induced by *Lipara* spp. flies (Tables 2 and 3). Of the species found in longitudinally cut galls, we only did not describe the nests of *Passaloecus clypealis* and *Hylaeus incongruus* due to the small number of nests (3 and 1 nests, respectively) in our dataset.

**Table 2:** Diameter of the reed stems and galls and the number of brood cells in the galls examined and in those containing the brood of species of aculeate Hymenoptera (Vespoidea, Apoidea) and their parasitoids (Hymenoptera: Chrysidoidea; Diptera: Asiloidea).

Species	Number of galls (nests) examined	Diameter of the reed stem [mm]		Diameter of the reed gall [mm]		Number of brood cells per gall	
		Mean ± SD	Range	Mean ± SD	Range	Mean ± SD	Range
**Diptera / Bombyliidae**							
*Thyridanthrax fenestratus*	18	3.4 ± 0.8	2–5	12.1 ± 2.0	9–18	5.6 ± 1.8	2–8
**Hymenoptera / Chrysididae**							
*Chrysis angustula*	1	N/A	4	N/A	10	N/A	5
*Trichrysis cyanea*	16	3.1 ± 1.0	2–5	9.2 ± 1.8	5–13	2.1 ± 1.4	1–5
**Hymenoptera / Vespidae**							
*Symmorphus bifasciatus*	6	3.7 ± 0.8	3–5	11.5 ± 3.6	8–16	3.5 ± 2.4	1–7
**Hymenoptera / Crabronidae**							
*Passaloecus clypealis*	3	2.3 ± 0.6	2–3	7.3 ± 1.5	6–9	2.3 ± 0.6	2–3
*Pemphredon fabricii*	1029	3.2 ± 1.0	1–7.5	10.0 ± 2.3	5–25	4.3 ± 1.8	1–12
*Trypoxylon deceptorium*	39	2.8 ± 0.7	2–5	9.1 ± 2.1	5–15	2.5 ± 1.5	1–6
*Trypoxylon minus*	6	2.8 ± 0.7	2–4	9.2 ± 1.0	8–10	3.2 ± 1.7	1–6
**Hymenoptera / Megachilidae**							
*Hoplitis leucomelana*	10	2.9 ± 0.9	2–4	9.0 ± 2.0	6–12	2.4 ± 1.1	1–4
**Hymenoptera / Colletidae**							
*Hylaeus incongruus*	1	N/A	2	N/A	10	N/A	3
*Hylaeus moricei*	3	3.7 ± 1.2	3–5	7.3 ± 1.5	6–9	4.3 ± 2.3	3–7
*Hylaeus pectoralis*	27	3.6 ± 1.2	2–6.5	10.7 ± 2.7	8–19	3.7 ± 2.1	1–11
**Not identified**	230	N/A		N/A		N/A	
**Unoccupied**	4629	3.7 ± 1.4	1–12	8.7 ± 3.0	2–23	N/A	
**TOTAL (reed galls examined)**	**6449**						

We examined only the old galls induced by the *Lipara* spp. at least one year prior the sampling.

**Table 3:** Species-specific occupancy rate of reed galls induced by *Lipara* spp. at least one year prior the sampling.

Species	Number of galls examined	Occupancy rate [%]								
		Diameter of the reed stem [mm]				Diameter of the reed gall [mm]				
		<4	4–5.5	≥6	Significance	<5	5–9.5	10–14.5	>15	Significance
**Diptera / Bombyliidae**										
*Thyridanthrax fenestratus*	18	0.35	0.30	0.00	>0.05	0.00	0.06	0.70	0.39	<0.001
**Hymenoptera / Chrysididae**										
*Trichrysis cyanea*	16	0.32	0.25	0.00	>0.05	0.00	0.30	0.38	0.00	>0.05
**Hymenoptera / Vespidae**										
*Symmorphus bifasciatus*	6	0.09	0.12	0.00	>0.05	0.00	0.09	0.05	0.78	0.005
**Hymenoptera / Crabronidae**										
*Passaloecus clypealis*	3	0.09	0.00	0.00	>0.05	0.00	0.09	0.00	0.00	>0.05
*Pemphredon fabricii*	1029	22.5	12.6	3.7	<0.001	0.00	14.1	23.7	18.8	<0.001
*Trypoxylon deceptorium*	39	1.07	0.21	0.00	<0.001	0.00	0.72	0.65	0.39	>0.05
*Trypoxylon minus*	6	0.13	0.08	0.00	>0.05	0.00	0.09	0.14	0.00	>0.05
**Hymenoptera / Megachilidae**										
*Hoplitis leucomelana*	10	0.22	0.13	0.00	>0.05	0.00	0.18	0.19	0.00	>0.05
**Hymenoptera / Colletidae**										
*Hylaeus moricei*	3	0.06	0.04	0.00	>0.05	0.00	0.09	0.00	0.00	>0.05
*Hylaeus pectoralis*	27	0.54	0.30	0.61	>0.05	0.00	0.39	0.60	0.39	>0.05
**Unoccupied**	4629	69.6	82.7	94.7	<0.001	95.4	81.4	67.4	75.0	<0.001
	**Number of galls examined**									
**TOTAL (reed galls examined)**	6449	3344	2515	506		281	3416	2381	287	

Total number of galls in each size category and their species-specific occupancy rates are indicated. The *p* values were obtained by χ^2^ tests testing the hypothesis that the occupancy rates were equally distributed among the size classes.

***Pemphredon fabricii*** (Fig 2A). The nests of the most abundant species in our dataset consisted of highly variable numbers of brood cells (range 1 to 12; median 4; mean 4.3 ± 1.8 cells per nest, n = 1029 nests). Brood cells (length 7.9 ± 1.9 mm) were separated from each other by 1–2 mm thick black-colored bars (septa) of unidentified material mixed with silk and larval salivae. This material might consist of the liquid larval feces greased on silk bars between the cells. We made this conclusion because no feces were found in brood cells and defecating younger larvae observed during the summer were extracting black liquid solution (P. Bogusch, unpubl.). Dry plant matter and soil particles were present in this substance, too. In some cases, behind the last cell and/or in front of the first cell, closing plug consisting of small sand grains mixed with very small pieces of plant tissues (leaves, stems of size around 1 mm) was used, but only very sparsely. Every nest had thin bar on the top consisting of the same black matter. The nests of *P*. *fabricii* were placed in galls induced prevalently at the very thin reed stems (up to 4 mm in diameter, at which 22.5% of galls were occupied. In contrast, *P*. *fabricii* occupied only 12.6% of galls induced at the reed stems of the diameter 4–5.5 mm, and just 3.7% of galls at thick reed stems of ≥6 mm in diameter (Table 3). However, the galls having less than 5 mm in diameter were completely avoided, and we found the highest occupancy rate in the galls of 10–14.5 mm in diameter (Table 3). Larvae pupated in the spring without any cocoon. Imagines of *P*. *fabricii* hatched usually before other aculeate hymenopteran species, 10–22 days after the pupation.

Larvae of *Trichrysis cyanea* were well recognizable in the nests of *P*. *fabricii*, because they pupated in funnel-like fibrous cocoons of red-brownish color (see Fig 2D and 2F). Also the larvae of *Chrysis angustula* pupated in brownish cocoons but their shape was cylindrical.

***Trypoxylon deceptorium*** **and** ***T***. ***minus***. Nests of *T*. *deceptorium* (Fig 2B) consisted usually of 1–2 brood cells, less frequently up to 6 cells (range 1–6; median 2; mean 2.5 ± 1.5 cell per nest, n = 39 nests). The brood cells were surprisingly large (length 8.3 mm ± 2.0 mm). Larvae were covered in silk cocoons of the light brown non-transparent color, with dark tough bars present in the front part of the cocoons. The pupation took part in the same cocoons. All bars between the brood cells and also the closing plug of the nest were made of mud. At (post)industrial sites (ash dumps of power stations), the grey mud made from the fly ash was used (Fig 2B). We have not found any feces in cocoons but think that black feces were glued in the back side of the cocoons and were recognized as the darkish tough bar. The nests of *T*. *deceptorium* were placed in galls induced prevalently at the very thin reed stems (up to 4 mm in diameter, at which 1.07% of galls were occupied by this species. In contrast, *T*. *deceptorium* occupied only 0.21% of galls induced at the reed stems of the diameter 4–5.5 mm, and was absent in galls at thick reed stems of ≥6 mm in diameter (Table 3). However, the galls having less than 5 mm in diameter were completely avoided, and we found the highest occupancy rate in the galls of 5–14.5 mm in diameter (Table 3). The larvae pupated early in the spring and the pupae had the typical *Trypoxylonini*-associated notches in their inner eye margin. Imagines hatched early, similarly to *Pemphredon fabricii*. The larvae of *Trichrysis cyanea* in nests of *T*. *deceptorium* (see Fig 2F) pupated similarly as in the nests of *P*. *fabricii*.

Nests of *Trypoxylon minus* displayed the identical structure and were impossible to distinguish from the nests of *T*. *deceptorium*. We identified six nests of this species (according to the individuals hatched from nests), with 1–4 brood cells (range 1–6; median 3; mean 3.2 ± 1.7 cell per nest, n = 6 nests). The portion of nests with more than two cells was higher than in *T*. *deceptorium*, but more nests would be needed to check whether these differences are statistically significant. This species had the biggest brood cells, length 8.5 mm ± 1.3 mm. The nests of *T*. *minus* were absent in galls induced at thick reed stems of ≥6 mm in diameter and in galls with maximum diameter ≤5 mm (Table 3).

***Hylaeus pectoralis*** (Fig 2E). Nests of *H*. *pectoralis* consisted usually of 1–5 brood cells (range 1–11; median 3; mean 3.7 ± 2.1 cell per nest, n = 27 nests). The nests usually did not comprise the whole gall. All nests of *Hylaeus* species were characteristic by their cellophane-like layers between the brood cells and also on the surface of the brood cells. These layers consist of a secret of female Dufour’s glands, which is used as a protection against pathogens. In some nests, the brood cells were separated also by a layer of small particles of reed leaves. The brood cells were 7.8 ± 2.0 mm long. The larvae were present inside the cellophane chambers with a very small bump of feces of nectar and pollen digested. The cavity in the gall behind the first built brood cell was usually filled with small cut parts of reed leaves of the size around 1–3 mm. Similar filling was used between the last built brood cell and closing plug of the nest, which was made of the same small parts of reed leaves but mixed probably with the secret of female’s Dufour gland. Bars between the brood cells were of highly variable thickness, from very thin (< 1 mm) to conspicuously thick ones (3–4 mm). The difference in the bar thickness depended on whether the plant material was incorporated in them. In contrast to the other reed gall inquilines, only the nests of *H*. *pectoralis* (and *P*. *fabricii*) were present in galls induced at thick reed stems of ≥6 mm in diameter (Table 3). Larvae pupated in brood cells without any cocoon, usually later than those of *P*. *fabricii* and *T*. *deceptorium*. Imagines hatched about 1–3 weeks later (after 21–39 days from the pupation) than adults of *P*. *fabricii*.

***Hoplitis leucomelana*** (Fig 2C). The nests of this species consisted of only 1–4 brood cells (median 2; mean 2.4 ± 1.1 cell per nest, n = 10 nests). The brood cells (length 8.2 ± 1.2 mm) were usually placed at the bottom of the gall cavity, and the rest of the gall was filled with a dry plant particles (usually reed leaves) and soil grains. The part filled by the debris was in some cases longer than the part with brood cells (see Fig 2C). Mature larvae were in brownish, semitransparent silk cocoons with darker back, separated by thin bars of brownish mixture of dry plant particles (probably mixed with soil grains and some other undefined particles). Closing plug of the nest was made of the same substance. The nests of *H*. *leucomelana* were absent in galls induced at thick reed stems of ≥6 mm in diameter and in galls with maximum diameter ≤5 mm (Table 3). Larvae pupated in their cocoons in similar time (21–35 days) as those of *H*. *pectoralis*.

***Symmorphus bifasciatus***. Nests of this species looked very similar to the nests of *Hoplitis leucomelana* (but without remains of pollen) and contained also similar number of cells (range 1–7; median 2.5; mean 3.5 ± 2.4 cell per nest, n = 6 nests). Larvae were in semitransparent cocoons made probably of silk mixed with undefined secret. These cocoons were for the first sight similar to those of *Hoplitis leucomelana* but were more transparent and of yellow or green color. The closing plug was made of chewed substance of soil and dry leaves; similar mixture was found in one nest above the last brood cell. The nests of *S*. *bifasciatus* were absent in galls induced at thick reed stems of ≥6 mm in diameter and in galls with maximum diameter ≤5 mm (Table 3). Larvae pupated in their cocoons little later than those of *P*. *fabricii*, first males emerged with *P*. *fabricii*, while other imagines reared later (19–37 days after the pupation). The length of brood cells was 7.8 ± 0.8 mm.

### Description of mature larvae

**Fig 4:**
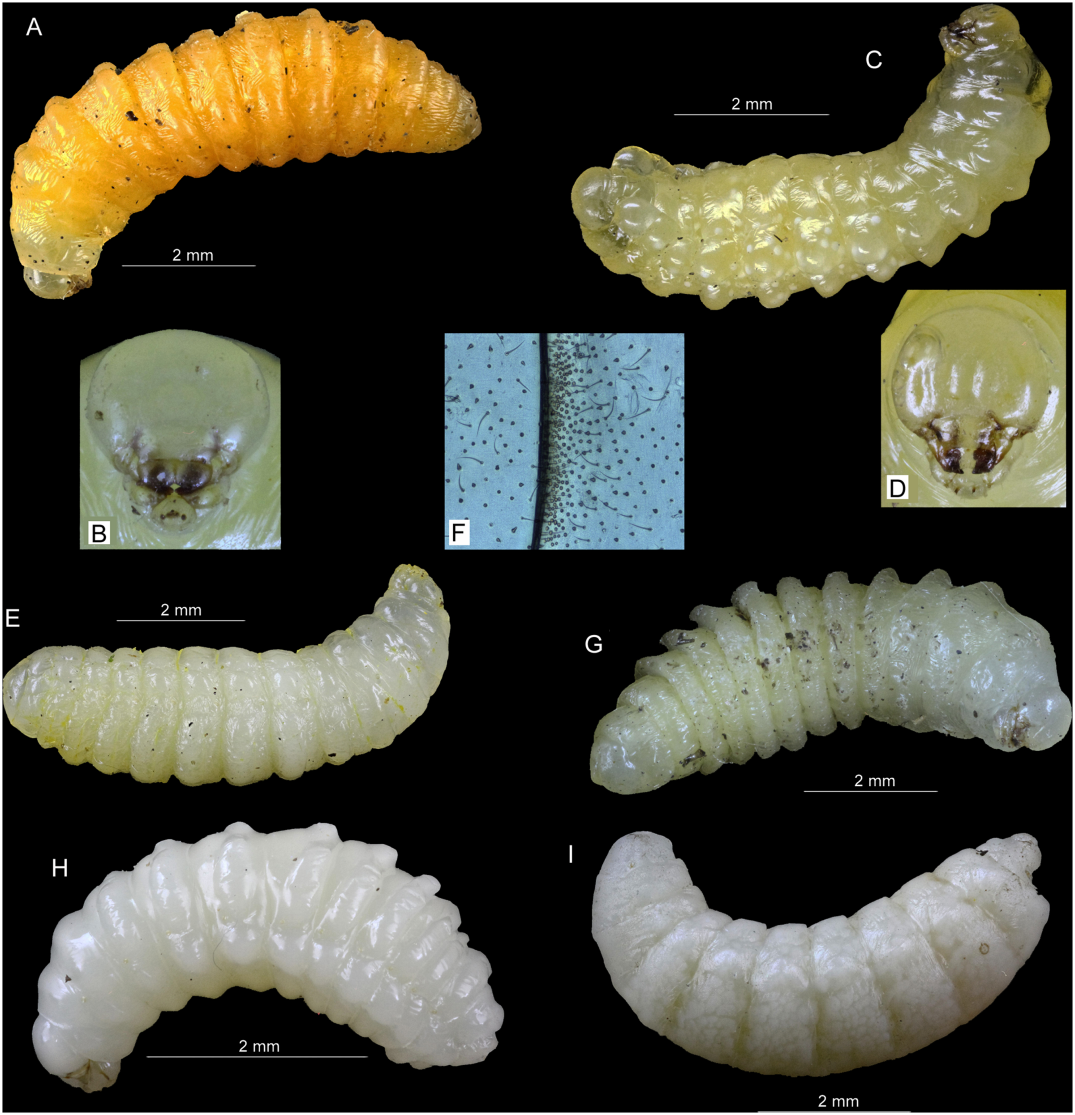
Larvae of aculeate Hymenoptera from nests in reed galls of *Lipara* spp. A—*Pemphredon fabricii*, whole larva, lateral view, B—*Pemphredon fabricii*, head, frontal view, C—*Trypoxylon deceptorium*, whole larva, lateral view, D—*Trypoxylon deceptorium*, head, frontal view, E—*Hoplitis leucomelana*, whole larva, lateral view, F—*Hoplitis leucomelana*, typical structure of setae and sensillae between body segments, G—*Hylaeus pectoralis*, whole larva, lateral view, H—*Trichrysis cyanea*, whole larva, lateral view, I—*Thyridanthrax fenestratus*, whole larva, lateral view. All photos by P. Bogusch.

**Fig 5:**
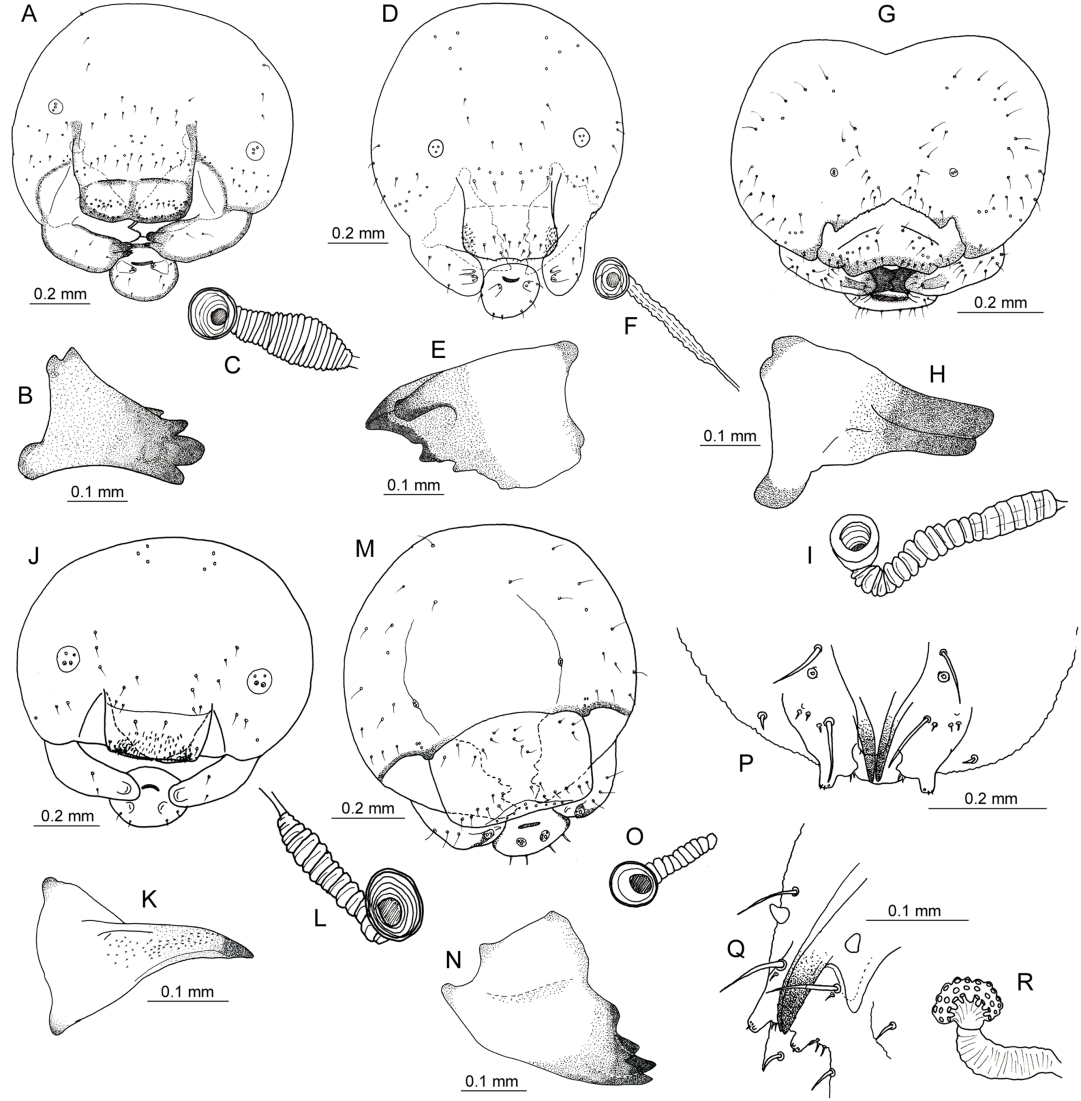
Larval characters. A—C—*Pemphredon fabricii*, A—head, frontal view, B—mandible, C—spiracle, D—F—*Trypoxylon deceptorium*, D—head, frontal view, E—mandible, F—spiracle, G—I—*Hoplitis leucomelana*, G—head, frontal view, H—mandible, I—spiracle, J—L—*Hylaeus pectoralis*, J—head, frontal view, K—mandible, L—spiracle, M—O—*Trichrysis cyanea*, M—head, frontal view, N—mandible, O—spiracle, P—R—*Thyridanthrax fenestratus*, P—mouthparts, frontal view, Q—mouthparts, lateral view, R—spiracle. All drawings by P. Bogusch.

We analyzed mature larvae of four species of Hymenoptera: Aculeata nesting in reed galls induced by *Lipara* spp., and two parasitoid species found in their nests (Tables 2 and 3). Below, we provide the descriptions of mature larvae, including the photos of whole larvae (Fig 4) and drawings of main determination characters (Fig 5).

***Pemphredon fabricii***. Mature larvae of *P*. *fabricii* were not previously described. The hitherto available descriptions of mature larvae of *P*. *lethifer* by [14–15] were not based on the material collected from reed stems or reed galls, thus the larvae described as *P*. *lethifer* sensu lato probably belonged to *P*. *lethifer* sensu stricto or some other newly established species of the *P*. *lethifer* complex. Here we provide the first description of mature larva of *P*. *fabricii*. Of note is that the larva of *P*. *fabricii* does not differ morphologically from the larva of *P*. *lethifer* described by the above mentioned authors, which is consistent with very close phylogenetic relationship of *P*. *fabricii* and *P*. *lethifer*.

Material: Czech Republic, Bohemia bor., Prunéřov, lignite power station fly ash deposit, 9 Mar 2014, 2 larvae, P. Heneberg lgt.; Slovakia, Slovakia occ., Sekule, terrestric reed bed surrounding little fishponds, 15 Mar 2014, 8 larvae, 4 Sep 2014, 8 larvae, P. Bogusch et A. Astapenková lgt., all P. Bogusch det. et coll.

Body. Length 8.3 ± 1.9 mm (n = 18), maximum width ~2 mm. Color yellow (most common), white or orange, in some cases reddish, or any shades of the above colors (light orange, light yellow, etc.); yellow or orange larvae possess frequently the first body segments (pronotum and part of mesonotum) white (Fig 4A). Posterior parts of segments form on dorsal part transverse welts slightly medially interrupted, continuous on the sides to pleural lobes, which are in some cases weakly developed. These structures are very ill formed on last three abdominal segments and on pronotum. Anus is a transverse slit, the supraanal lobe is produced. Spiracles small, light brown, first pair slightly larger than other nine pairs, atrium very weakly marked with five lines, subatrium unarmed (Fig 5C). Integument smooth, not hairy, only with a few weak setae on the dorsum of each body segment.

Head and mouthparts. Head distinctly visible, not protruded or embedded into the prothorax, slightly narrower than prothorax, width 0.962 ± 0.012 mm, height 0.856 ± 0.02 mm, width: height ratio >1. Pale and unpigmented except for brownish markings at the following positions: anterior and posterior tentorial arms, pleurostomal and hypostomal thickenings, mandibles, entire margin and a medial band on the labrum, latero-basal margin and a subapical ring on the maxilla, a circular ring on the praementum, palpi and galeae (Figs 4B and 5A). Antennal orbits large, circular, with three sensory cones in the membrane. Head with a few punctures, most of them anteriorly positioned from the orbits, clypeus with a broad band of punctures with setae. Labrum bilobed, about three times wider than long, with sclerotized and pigmented marginal and median bands, numerous punctures in the apical half, 14 of them with setae, the other with small sharp projection, several sensilla near the margin, epipharynx with numerous small spinulae. Mandibles (length 0.34 ± 0.009 mm) with four apical teeth, the basal is smallest, two lateral teeth more or less one above the others, only one single lateral basal setae on the mandibles (Fig 5B). Maxillae with some strong lateral setae, palpi stout and blunt with five apical sensillae, galeae smaller. Labial palpi long with four sensillae, spinerettes form a well visible salivary slit, hypopharynx with spinules.

***Trypoxylon deceptorium***. The species *T*. *attenuatum* was divided into six separate species [23]. *T*. *deceptorium* is a thermophilous wetland species, quite common in most of south and central Europe [17]. Asís et al. [22] described mature larva of *T*. *attenuatum* but the description of the biology suggests that their description could in fact represent *T*. *deceptorium*. Here we provide the first description of mature larva of *T*. *deceptorium* identified to the species level according to the current nomenclature. The described larva differs only slightly from that described [22] in the number of mandibular teeth, which could simply represent an unintentional error present in the previously published description.

Material: Czech Republic, Moravia bor., Stonava, wetland at a depression caused by underground black coal mining partially filled with tailings, 7 Feb 2014, 3 larvae, P. Heneberg lgt., Slovakia, Slovakia occ., Sekule, terrestric reed bed surrounding little fishponds, 4 Sep 2014, 9 larvae, P. Bogusch lgt., all P. Bogusch det. et coll.

Body. Length 5.5 ± 1.6 mm (n = 12), subcylindrical shape, white or pale yellowish (ochre) colored. Posterior parts of segments with distinct lobes, pleural lobes also well developed, they are weaker on prothorax and most distinct on last abdominal segments, which are also the widest (Fig 4C). Anus ventral, last segment rounded, with two spine-like processes on the sides. Spiracles pale brown, very small, atrium very weakly marked with five lines, subatrium unarmed (Fig 5F). Body integument very thin and smooth, with few setae especially on the dorsum and pleural lobes.

Head. Head well developed, slightly wider than long (width 0.811 ± 0.013 mm, height 0.799 ± 0.01 mm), narrower than prothorax. Pale and unpigmented except the following structures: pleurostomal and hypostomal thickenings, anterior tentorial arms, mandibles, margins of praementum, epipharynx, apical part of laccinia, maxillar palpus and galea, praementum, salivary slit and labial palpus (Figs 4D and 5D). Antennal orbits large, circular, with three sensory cones in the membrane. Head with only a few punctures, most of them bearing setae (three on each side of the head and majority on the labrum). Clypeus with 6 setae and 6 sensillae, rectangular shape, unsclerotized. Labrum unsclerotized with 12 setae and several sensillae in the middle of the apical margin, which forms a very shallow depression, and two small unsharp teeth on each side of this depression. Postero-lateral parts of epipharynx bear plenty of short, sharp tooth-like processes. Mandible (0.269 ± 0.005 mm long) with six teeth, one apical, three lateral, one basal and one on the distal side (Fig 5E). Maxillae well developed with sclerotized palpi bearing three sensillae, which are larger than galeae, lacciniae with a spinulous lobe. Labium with short palpi with one sensilla and salivary slit, all sclerotized. Hypopharynx ill visible, unpigmented, with small spinules.

***Trichrysis cyanea***. This very common species is a parasitoid in nests of many other aculeate hymenopterans and its larvae were described [22] from the nests of *Trypoxylon attenuatum* s. l. Our description is similar with their and show that also larvae of *T*. *cyanea* from nests of *Pemphredon fabricii* have the same morphology.

Material: Slovakia, Slovakia occ., Sekule, terrestric reed bed surrounding little fishponds, 4 Sep 2014, 2 larvae, P. Bogusch lgt., det. et coll.

Body. Short and robust, white-colored, length 3.8 and 4.0 mm (n = 2). Shape fusiform with well-developed dorsal posterior lobes reaching the pleurae of the segments. Pleural lobes well developed and forming a line (Fig 4H). Last abdominal segment very short and narrower than other segments. Anus terminal as a transverse slit. Integument with microspinules, especially on dorsal anterior parts of the terga, setae only in very small number. Spiracles pale brown, quite big, atrium very weakly marked with five lines, subatrium unarmed (Fig 5O).

Head. Head quite big but narrower than prothorax, only slightly wider than long (width 0.874–0.899 mm, height 0.825–0.852 mm), most of the head pale, unpigmented. Sclerotized and pigmented are the following structures: pleurostomal and hypostomal thickenings, mandibles, mandibular condyli and genae, apical parts of labrum, maxillae and labium (Fig 5M). Anterior orbits very small, irregularly oval, with two sensory cones in the membrane. Setae in a low number on the whole surface of the head, more very long setae on the sides near mandibular condyli. More setae on apical part of labrum and maxillae. Clypeus rectangular with 18 punctures bearing setae, most of them in basal part. Labrum roundly rectangular with 18 punctures with setae and some sensillae mainly in the basal part. Epipharynx relatively smooth, apically with a row of 14 big sensillae. Mandibles large (length 0.0290–0.291 mm), well sclerotized, with five prominent teeth, without setae (Fig 5N). Maxillae with three prominent setae on lateral part of the laccinia, maxillar palpi short with four sensillae, galeae very narrow. Labium with short palpi bearing five sensillae (one of them bigger than the other) and long, transverse salivary slit. Hypopharynx rugose and well sclerotized.

***Hoplitis leucomelana***. The larva of this common bee was described by [21], but the description is insufficient and available only in a poorly available publication. The morphology described fits well the central European specimens of this species.

Material: Czech Republic, Moravia mer., Hodonín env., heating plant slag/ash deposit, 14 Mar 2014, 2 larvae, P. Bogusch et A. Astapenková lgt., Slovakia, Slovakia occ., Sekule, terrestric reed bed surrounding little fishponds, 4 Sep 2014, 4 larvae, P. Bogusch lgt., all P. Bogusch det. et coll.

Body. Robust, white-colored, length 8.1 ± 0.5 mm (n = 6). Shape fusiform with ill developed dorsal posterior lobes on the segments (Fig 4E). Anus terminal as a transverse slit, last abdominal segment rounded. Integument bearing plenty of setae and spinules, especially on lateral parts of tergites (Fig 4F). Very rich concentration of spinulae is near posterior margins of abdominal segments, those ochre or yellowish colored. Spiracles pale brown, smaller, atrium round and widely marked with 7 lines, subatrium unarmed (Fig 5I).

Head. Well-developed but small, wider than long (width 0.788 ± 0.01 mm, height 0.692 ± 0.009 mm), heart shaped, very much narrower than prothorax. Pale, unpigmented, except the following structures: pleurostomal and hypostomal thickenings, anterior tentorial arms, labrum (especially the posterior part), mandible, posterior part of maxillae with galea and palpus, labium with salivary slit and palpus (Fig 5G). Clypeus very weakly sclerotized. Antennal orbits small, circular, unsharply marked, with one sensory cone in the membrane. Head with many punctures bearing setae and with spinules, the concentration of these structures is similar all over the shape. Clypeus V-shaped, very slightly sclerotized, with 10 punctures bearing setae. Labrum triangular with wide concave depression, labral margin thick a pugged with rough structure on the epipharynx. Labrum laterally with 16 punctures with sensillae (8 on each side) and 18 without (9 on each side), several microsensillae on the anterior margin in the middle. Mandible (length 0.243 ± 0.003 mm) with two teeth, sclerotized and without sensillae (Fig 5H). Maxillae with elongated palpus longer than galea with two sensillae on the top, laccinia with 13 punctures with setae on lateral part. Labium with very well developed salivary slit in the middle, palpus elongated with two sensillae. Hypopharynx brownish, sclerotized and rugous.

***Hylaeus pectoralis***. Janvier [18] described the nest and larva of this species in France. Morphology of here described central European specimens did not differ from the original description but more details and more detailed figures are provided.

Material: Czech Republic, Bohemia mer., Lomnice nad Lužnicí, Velký a Malý Tisý National Natural Reserve, terrestric reed bed surrounding a fishpond, 22 Feb 2014, 3 larvae, Bohemia mer., Třebeč, Brouskův mlýn National Natural Reserve, terrestric reed bed surrounding the river, 21 Feb 2014, 2 larvae, both P. Heneberg lgt., Slovakia, Slovakia occ., Sekule, terrestrial reed bed surrounding little fishponds, 4 Sep 2014, 1 larva, P. Bogusch lgt., all P. Bogusch det. et coll.

Body. White-colored, length 7.3 ± 1.0 mm (n = 6), shape fusiform with dorsal posterior lobes well developed and extending to the pleurae, these structures are missing on prothorax and mesothorax and on last four abdominal segments (Fig 4G). Anus terminal as a transverse slit, last abdominal segment rounded. Integument smooth with only very few setae on dorsal part. Spiracles with narrowly rounded atrium with seven lines, subatrium unarmed (Fig 5L).

Head. Well developed, wider than long (width 0.912 ± 0.014 mm, height 0.822 ± 0.01 mm), transparent, sclerotized only on the following parts: pleurostomal and hypostomal thickenings, mandibles, apical part of clypeus, labium and maxillae only slightly sclerotized (Fig 5J). Orbits round, big, with four sensory cones in the membrane. Head with only few sensillae, most of them with setae. Clypeus rectangular, wide, with only two sensillae bearing setae on each side. Labrum of similar shape but longer, with punctures bearing very tough setae on the sides and many setae without punctures on the surface, especially in posterior part, and 12 punctures bearing setae in various parts of the labrum. Epipharynx with very rough structure. Mandible (length 0.354 ± 0.016 mm) with one tooth, sharp, with many little spinules on inner lateral side, inner apical part without toothlike processes (Fig 5K). Maxillae elongated and unsharp, maxillar palpi rounded without visible sensillae. Labium with very small round palpi and sclerotized salivary slit in the centre. Hypopharynx well visible and rough.

***Thyridanthrax fenestratus***. The larva of this common dipteran parasitoid of various digger wasps was described by [30]. However, the hitherto available description is very short and lacks good drawings. We compared the larvae from nests both of *Pemphredon fabricii* and *Trypoxylon deceptorium* and found they look similar to each other.

Material: Slovakia, Slovakia occ., Sekule, terrestric reed bed surrounding little fishponds, 15 Mar 2014, 7 larvae, 4 Sep 2014, 5 larvae, P. Bogusch et A. Astapenková lgt., all P. Bogusch det. et coll.

Body. White-colored with matt appearance, 4.9 ± 0.8 mm long (n = 12). Body segments smooth without processes or lobes, posterior parts only slightly protruding ventrally (Fig 4I). Last abdominal segment narrower with very narrow apical part, which is rounded with circular anus. Spiracles only on the pronotum and posteriorly on the anterior part of last abdominal segment, atrium of abdominal spiracle weakly marked but with several wide lines of rosettes (Fig 5R). Integument strong with rough structure on the whole surface, without any setae. Anterior end of the body wide, head indistinct.

Head unsclerotized except the mouthparts, which are also brownish pigmented. Mandibles prominent, sharplike with three teeth oriented backwards and well visible basal lobe, antennae short but well visible, consisting of two segments. Labrum on dorsal part, trapezoidal, well sclerotized. Maxillae lobe-like formed, with elongated two segmented palpi bearing three (two elongated and one flat) sensillae. Labium small, indistinct. Several setae around the mouthparts present, two on each side very big, prominent (Fig 5P and 5Q).

## Discussion

Recent research in landscape ecology [31–32] and natural history and taxonomy [27, 33] extensively benefited from the use of trap nests, including the trap nests made from the common reed stems and galls, which are now frequently used to collect solitary cavity-nesting Hymenoptera and their parasitoids. However, the data from the reed stems and galls examined *in situ* (without constructing the trap nest itself) are scarce. Thus, this study improves not only our knowledge on the larvae and nests of the reed gall-associated species, which could be potentially found in such trap nests, but also address the species composition associated with such nesting resources. Combined data ([2–4] and this study) suggest that the assemblage of aculeate Hymenoptera nesting in reed galls induced by *Lipara* flies comprises in Europe at least 29 species of nesting bees and wasps, 2 cleptoparasites of the genus *Stelis* and 4 parasitic golden wasps bound on their nests, including several facultative reed gall inquilines newly identified in this study. The reed galls host a broad spectrum of rare species. Among them are *Passaloecus clypealis*, *Rhopalum gracile*, *Hylaeus moricei* [3] and this study, *Stenodynerus xanthomelas* [2] and *Ectemnius confinis* [4], all nesting in reed galls as well as reed stems. *Stenodynerus xanthomelas*, recorded by [2], is a very rare species of wet meadows and its ecology seems to be similar to *R*. *gracile* and other species mentioned in this paragraph, but is probably absent in the Czech Republic [16]. Of interest is the record of *Heriades rubicola*. This species is currently expanding to the north and reached the southernmost parts of the Czech Republic only a few years ago. The first published records in south Moravia date back to the year 2012 [34] but the first finding was made by J. Straka (unpubl.) in Tasovice (ZN, Czech Rep.) already in 2007. Recently, it is increasingly common at sites with the presence of common reed in southernmost Moravia, and here we confirmed that it nests in reed stems and in galls induced by *Lipara* spp.

Only three species found in the reed galls could be considered as common. *Pemphredon fabricii* feeds on reed aphid secrets and does not fly away from the reed beds [11]. It is probably dependent on galls induced by the *Lipara* flies as the only nesting resource [2–3] (determined as *P*. *lethifer* sensu lato in the reference by Westrich). *Hylaeus pectoralis* prefers reed galls as well but it occurs at a narrower spectrum of sites compared to *P*. *fabricii* and it is also less abundant at sites of its occurrence. However, by the analysis of the reed galls ([3] and this study), we showed that *H*. *pectoralis* is more common than was previously thought [3, 16, 35]. Of note is that we have not recorded other wetland species of the genus *Hylaeus* (except *H*. *moricei*), although *H*. *rinki* was found in color pan traps at the same sampling sites from where the reed galls were collected [3]. The last abundant species, *Trypoxylon deceptorium*, is a very common wetland specialist, which uses reed stems and perhaps some other cavities in addition to the galls induced by *Lipara* spp. for its nesting. Acceptance of multiple types of cavities by *T*. *deceptorium* is supported by the results of color pan trapping, which yielded more specimens than rearing the reed galls at sites where both these methods were applied [3]. The digger wasps of the genus *Trypoxylon* forage on flower nectar, so (in contrast to *P*. *fabricii* feeding mainly on aphid honeydew) its abundance can be effectively determined based on both the above methods. In this study, we found that these species may form mixed nests. In all such nests, brood cells of one species were made first, and then followed by the second, so the nesting females probably did not meet each other.

Most of the newly identified reed gall inquilines use reed galls only occasionally and nest also in other cavity types. Such behavior is characteristic for, e.g., common solitary wasp *Symmorphus bifasciatus*, digger wasps *Nitela spinolae* and *Trypoxylon minus* and bees *Pseudoanthidium lituratum*, *Chelostoma campanularum*, both *Stelis* spp., *Hylaeus incongruus* and *Hoplitis leucomelana*. Nartshuk and Andersson [4] published records on other species, however, without detailed source references. Some of them behave similarly to the above-mentioned species, occur in many kinds of habitats and nest in different cavity types. However, a couple other species published by [4] are unlikely to nest in galls induced by *Lipara* spp. and were probably misidentified. Among them was *Rhopalum clavipes*, which is a species of forests nesting in stems of plants [11] misidentified probably with *R*. *gracile*, the morphologically similar reed stem & gall specialist [3]. Also the records of other species of *Pemphredon* (*P*. *inornata*, *P*. *lethifer*, *P*. *rugifer* and *P*. *wesmaeli*), *Passaloecus* (*P*. *corniger*, *P*. *gracilis* and *P*. *singularis*) and *Trypoxylon figulus* are likely based on misidentification. But, contrary to *R*. *clavipes*, these species could probably occasionally nest in reed galls. In course of this and our previous study [3], we have checked thousands of individuals of *Pemphredon* from reed galls collected at dozens of sampling sites, and all represented *P*. *fabricii*, not the other species of *P*. *lethifer* complex.

Reed galls have limited space for nesting but the same situation is in the case of reed stems and most of the other cavities, too. There are also huge differences in the size of reed galls occupied by aculeate hymenopterans, some of them providing more than 15 cm long cavity but the others being shorter than 3 cm, which may in part explain the differences in the number of brood cells found. However, many nests comprised only a part of the cavity in the gall, and we found also nests extending to the soft top of the gall in contrary (and they were not rare). The species-specific size of the nest cells was not related to the size of the gall or larvae: for example mature larvae of *Trypoxylon minus* were shorter and smaller than those of *Hylaeus pectoralis* or *Pemphredon fabricii*, but the length of *T*. *minus* brood cells was the largest. In this study, we confirmed that most of the species prefer the wider galls but induced at thin stalks over the other types. Such galls are produced by *Lipara lucens* while other species of *Lipara* cause galls that are much narrower and not so conspicuous [4, 33, 36]. Galls of *L*. *lucens* have also very tough walls in contrary to the galls of other *Lipara* spp., and thus could serve as a good defensive structure against the predators and parasitoids.

Most of the species analyzed in this study used the reed leaves (cut to pieces or chewed and mixed with some other materials) to construct the septa between brood cells and other parts of the nest (closing plug or interspace fillings). Overall, the nest structures and materials used by the examined species resembled those used by the closely related species [14, 17, 20, 37, 38]. Similarly, the mature larvae were morphologically similar to the related taxa and to the descriptions of representative larvae of the current species complexes. Of note, the original descriptions [14, 15, 18, 21, 22, 37] paid little attention to the chaetotaxy (number and position of setae and sensillae on the body), which, however, should be considered as a good tool allowing the identification of the species.

The mature larva of *Pemphredon fabricii* is very similar to the larva of *P*. *lethifer*, as described in [14–15]. It shows similar total length, morphology of the body, coloration and the mouthparts, which is actually characteristic for altogether 10 species of the genus *Pemphredon* as described by [14, 37]. According to Janvier [14], the morphology of mandibles displayed the most striking differences between the species analyzed—larvae of all species have four mandibular teeth but their size and position is species-specific. Most prominent differences can be found when comparing the larvae of different subgenera, such as *P*. *morio* of subgenus *Ceratophorus* (with different size and number of mandibular teeth and also shape of the clypeus) with the subgenus *Cemonus* (*P*. *lethifer* and *P*. *fabricii*), which have mandibular teeth of similar size and all located near the apex of the mandible. However, we cannot compare the number of sensillae and setae on the head with other descriptions because there were only few remarks provided by [15] but we assume that the differences are probably minute. There are also small differences in the number of setae (± 1–2) on each part of the head among 10 larvae used for the description in this study. Thus, the main difference between *P*. *lethifer* and *P*. *fabricii* larvae consist of their mandibular teeth, where the smallest tooth on the inner side of the mandible is moved more laterally at *P*. *fabricii* than in *P*. *lethifer*.

Mature larva of *Trypoxylon deceptorium* corresponds very well to that of *T*. *attenuatum* described by [22], which was quite surprising. The authors of the description determined identification characters allowing to distinguish between the two subgenera of this genus, *Trypoxylon* and *Trypargilum*, which also allow to compare the larvae of European *Trypoxylon* spp. Because Asís et al. did not distinguish the taxa newly described by [23] from *T*. *attenuatum* sensu lato, they most likely described larvae *of T*. *deceptorium* species. Thus, in the future, it will be necessary to re-describe the larvae of *T*. *attenuatum* sensu stricto as well. Reed galls, however, host only *T*. *deceptorium*. Larvae of both species are expected to be very similar, because also the adults of both species are very similar and difficult to identify, and differ more ecologically than morphologically. Whereas *T*. *attenuatum* is a widespread species of various habitats, *T*. *deceptorium* occurs typically in wetlands and is more common in southern and warmer parts of Central Europe [17]. We found only one difference in the number of mandibular teeth. However, it is possible that [22] only did not notice the smallest tooth or did not mark it as tooth but as a projection or angle. In the nests of *T*. *deceptorium* and *P*. *fabricii*, we found the larvae of the parasitoid *Trichrysis cyanea*. The description of the larva of *Trichrysis cyanea* corresponds with that published by the same authors and larvae and imagines of *T*. *cyanea* from nests of both host species are morphologically similar to each other.

Mature larvae of *Hylaeus pectoralis* corresponded well with the description provided [18]. Yet unexplained is the profound reduction of rough structures at the apex of the larval mandible. We speculate that they could be considered as a result of adaptation for different food but data from other wetland *Hylaeus* spp. are not available so far. Similar situation is with *Hoplitis leucomelana*. The larva of this small member of the genus differs from the other species in the body size and also in the shape of clypeus and labrum. Most of the described larvae of this genus have two mandibular teeth similarly to *H*. *leucomelana* and the teeth are usually very similar in size [20].

The mature larva of *Thyridanthrax fenestratus* was already described by [30], and its drawing was published also by [39]. They found that the *T*. *fenestratus* mandibles are bidentate, with lateral hook. However, these descriptions are very different from the specimens analyzed in this study and differ also from the features of the whole family Bombyliidae. In general, the larvae of the family Bombyliidae have rough body integument and head reduced only to the mouthparts. Their mandibles are sharp and elongated, with three or four opposite spines. Maxillae and labrum look similar, and labrum has five small processes on the margin [39]. We have observed the structures similar to the above features shared with other bee flies, but not those described specifically in the larvae of *T*. *fenestratus* by the previous authors despite we prepared all mouthparts separately (see Fig 5P and 5Q for anterior and lateral views of the mouthparts). We also confirmed two new hosts of this bee fly, which was previously recorded as a parasitoid of digger wasps of the genera *Ammophila* and *Sphex* [39, 40]. In conclusion, using an extensive dataset of experimentally hatched reed galls, we elucidated the nesting biology and ecology, and provide the descriptions of larvae of aculeate hymenopterans nesting in reed galls and their parasitoids. Obtained data allow for the first time to identify the larvae of the most widespread central European aculeate hymenopteran reed gall specialists.

## Supporting Information

S1 Table List of the study sites. Localities with permission needed are marked by “Y” in the column “Permit”.
(DOCX)
