# Supplementary material for: Larvae and Nests of Six Aculeate Hymenoptera (Hymenoptera: Aculeata) Nesting in Reed Galls Induced by Lipara spp. (Diptera: Chloropidae) with a Review of Species Recorded
Source: PLoS One. 2015 Jun 26;10(6):e0130802. doi: 10.1371/journal.pone.0130802 (PMC4482587; doi:10.1371/journal.pone.0130802)
Supplement: S1 Table — Localities with permission needed are marked by “Y” in the column “Permit”. (DOCX) [file pone.0130802.s001.docx]

| **Sampling site** | **Township** | **District** | **Coordinates** | **Altitude** | **Sampling date** | **Permit** |
| --- | --- | --- | --- | --- | --- | --- |
| Darkov | Darkov | Karviná | 49.83N, 18.56E | 249 | 07-Feb-2014 |  |
| Stonava | Stonava | Karviná | 49.81N, 18.51E | 249 | 07-Feb-2014, 08-Feb-2014 |  |
| Doubrava u Orlové | Doubrava | Karviná | 49.86N, 18.49E | 238 | 08-Feb-2014 |  |
| Zlaté Hory v Jeseníkách | Zlaté Hory | Jeseník | 50.23N, 17.41E | 551 | 08-Feb-2014 |  |
| Hrušov | Ostrava | Ostrava-město | 49.87N, 18.31E | 208 | 09-Feb-2014 |  |
| Bohumín | Bohumín | Karviná | 49.88N, 18.35E | 201 | 09-Feb-2014 |  |
| Dubno | Dubno | Příbram | 49.69N, 14.06E | 510 | 16-Feb-2014 |  |
| Dolní Beřkovice | Dolní Beřkovice | Mělník | 50.40N, 14.39E | 208 | 17-Feb-2014 |  |
| Tišice | Tišice | Mělník | 50.28N, 14.53E | 164 | 17-Feb-2014 |  |
| Vašírov | Lány | Rakovník | 50.14N, 13.94E | 414 | 20-Feb-2014 |  |
| Stará Pohůrka | Srubec | České Budějovice | 48.96N, 14.52E | 432 | 20-Feb-2014 |  |
| Třebeč | Borovany | České Budějovice | 48.88N, 14.70E | 452 | 21-Feb-2014 | Y |
| Mydlovary u Dívčic; Olešník | Mydlovary; Olešník | České Budějovice | 49.09N, 14.36E | 409 | 21-Feb-2014 |  |
| Dívčice | Dívčice | České Budějovice | 49.10N, 14.33E | 395 | 21-Feb-2014 |  |
| Lomnice nad Lužnicí | Lomnice nad Lužnicí | Jindřichův Hradec | 49.06N, 14.74E | 427 | 22-Feb-2014 | Y |
| Kamenné Žehrovice | Kamenné Žehrovice | Kladno | 50.13N, 14.00E | 403 | 01-Mar-2014 |  |
| Všebořice | Ústí nad Labem | Ústí nad Labem | 50.70N, 13.97E | 232 | 01-Mar-2014 |  |
| Dělouš | Ústí nad Labem | Ústí nad Labem | 50.71N, 13.96E | 228 | 02-Mar-2014 |  |
| Tuchomyšl | Ústí nad Labem | Ústí nad Labem | 50.65N, 13.98E | 151 | 02-Mar-2014 |  |
| Kostomlaty pod Milešovkou | Kostomlaty pod Milešovkou | Teplice | 50.55N, 13.85E | 382 | 02-Mar-2014, 09-Mar-2014 |  |
| Hořany | Most | Most | 50.49N, 13.58E | 252 | 09-Mar-2014 |  |
| Prunéřov | Kadaň | Chomutov | 50.43N, 13.27E | 374 | 09-Mar-2014 |  |
| Náchod | Náchod | Náchod | 50.43N, 16.15E | 375 | 22-Feb-2014 |  |
| Dvůr Králové nad Labem | Dvůr Králové nad Labem | Trutnov | 50.42N, 15.82E | 288 | 22-Feb-2014 |  |
| Rybitví | Rybitví | Pardubice | 50.05N, 15.70E | 223 | 24-Feb-2014 |  |
| Srnojedy | Pardubice | Pardubice | 50.05N, 15.71E | 223 | 24-Feb-2014 |  |
| Chvaletice | Chvaletice | Pardubice | 50.03N, 15.43E | 224 | 07-Mar-2014 | Y |
| Trnávka | Chvaletice | Pardubice | 50.04N, 15.45E | 212 | 07-Mar-2014 | Y |
| Hodonín | Hodonín | Hodonín | 48.88N, 17.06E | 180 | 14-Mar-2014 |  |
| Lednice | Lednice | Břeclav | 48.79N, 16.82E | 159 | 14-Mar-2014, 15-Mar-2014 | Y |
| Sekule | Sekule | Skalica | 48.62N, 17.00E | 149 | 15-Mar-2014, 04-Sep-2014 |  |
| Hradec Králové | Hradec Králové | Hradec Králové | 50.21N, 15.85E | 229 | 09-Mar-2014 |  |
| Cheb | Cheb | Cheb | 50.10N, 12.25E | 444 | 22-Mar-2014 |  |
| Vonšov | Skalná | Cheb | 50.15N, 12.40E | 436 | 22-Mar-2014 | Y |
